# Supplementary figures and images for: TLR5 agonist entolimod reduces the adverse toxicity of TNF while preserving its antitumor effects
Source: PLoS One. 2020 Feb 6;15(2):e0227940. doi: 10.1371/journal.pone.0227940 (PMC7004342; doi:10.1371/journal.pone.0227940)

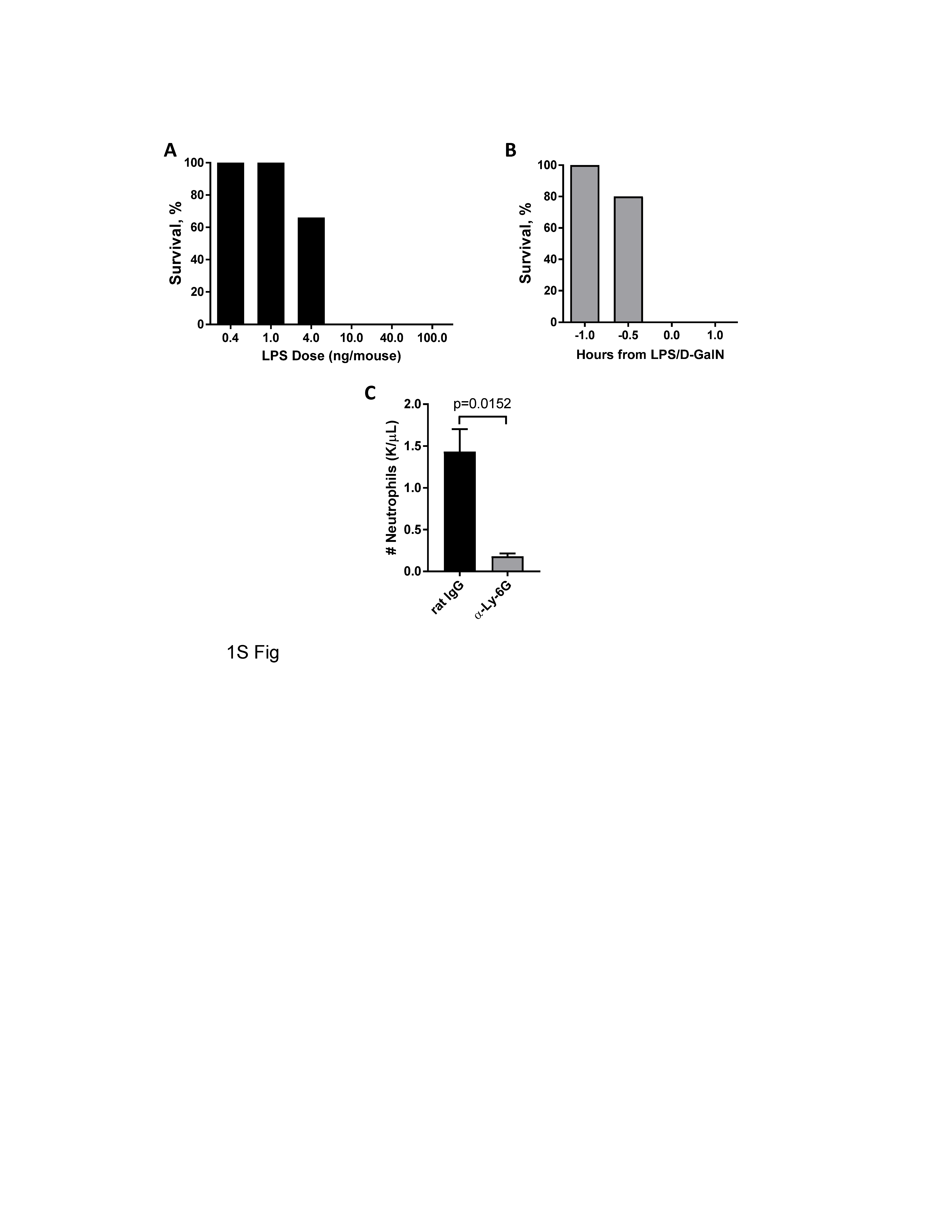

Supplement: S1 Fig — (A) Survival of BALB/c mice after injection of the indicated doses of LPS and D-GalN (16 mg/mouse). Mice were monitored for 2 weeks following treatment. All mortality occurred within 24 h after treatment (6 mice/group). (B) Survival (>48 h) of BALB/c mice after LPS (10 ng/mouse) and D-GalN (16 mg/mouse) treatment with a single dose of entolimod (1 μg/mouse) injected s.c. 30 min and 1 h before, simultaneously with (<1 min apart), or 1 h after LPS (5 mice/group). (C) Efficiency of neutrophil depletion in mice injected twice (24 h apart) with α-Ly6G antibody or an isotype-matched control rat IgG antibody. Neutrophil numbers were determined by complete and differential blood cell count (CBC) analysis of blood samples collected 1 h after the second antibody injection (3 mice/group). (TIF) [file pone.0227940.s001.tif]

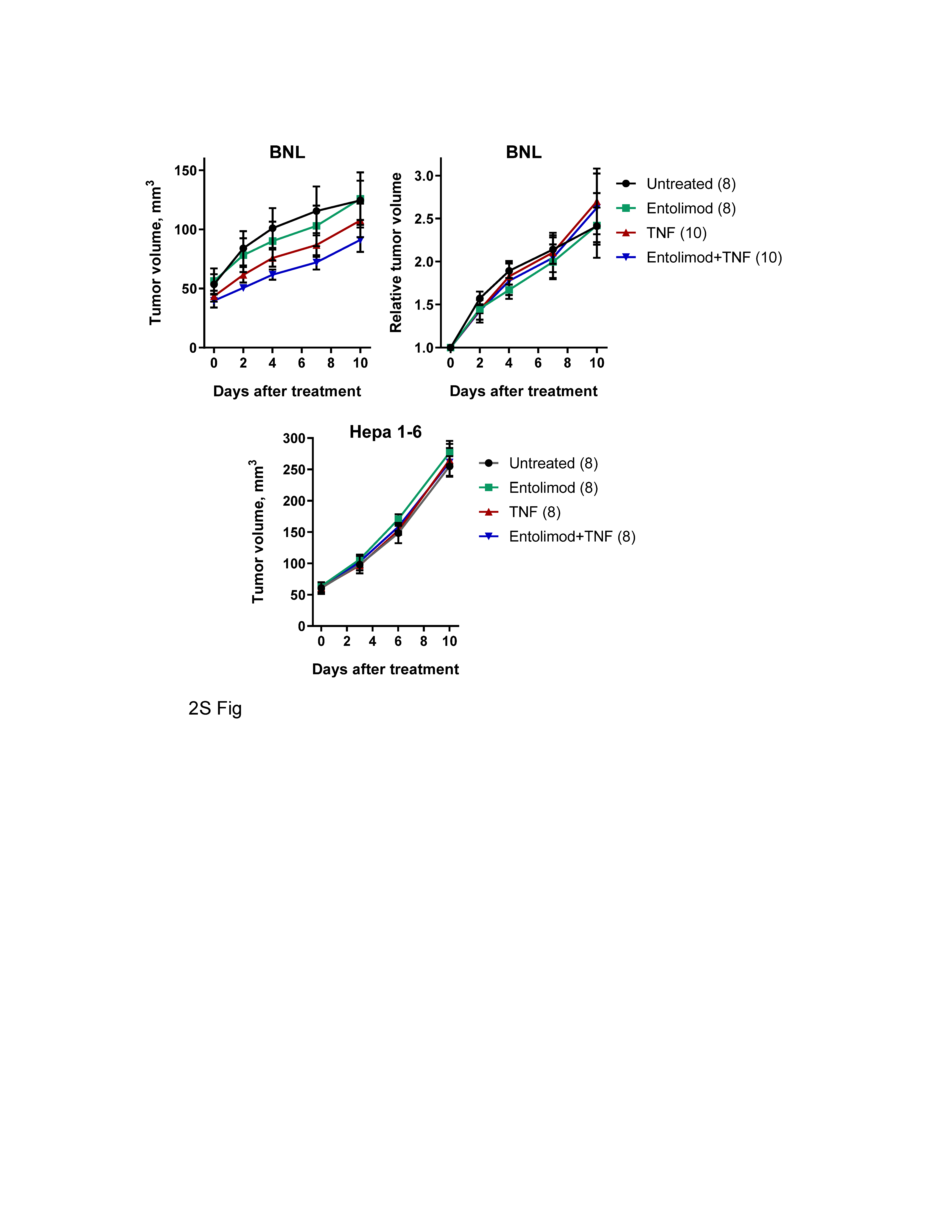

Supplement: S2 Fig — Mice were treated on day 0. Tumors were measured on the indicated days after treatment. Relative tumor volume on each day of measurement was calculated as a ratio to the tumor volume on the day of treatment (Day 0). Mean ±SEM is shown for the number of tumors per group indicated in parentheses (2 tumors per mouse). Control groups received entolimod alone or PBS (untreated). (TIF) [file pone.0227940.s002.tif]
