# Supplementary material for: TLR5 agonist entolimod reduces the adverse toxicity of TNF while preserving its antitumor effects
Source: PLoS One. 2020 Feb 6;15(2):e0227940. doi: 10.1371/journal.pone.0227940 (PMC7004342; doi:10.1371/journal.pone.0227940)
Supplement: S1 Table — (DOCX) [file pone.0227940.s003.docx]

**S1 Table. Genes upregulated by entolimod and/or LPS in livers of C57BL/6 mice**

| **Gene** | **Untreated** | **Entolimod** | | **LPS** | |
| --- | --- | --- | --- | --- | --- |
|  | **Mean** | **Mean** | **Fold change** | **Mean** | **Fold change** |
| ***CXCL1*** | 39.84 | 10243.56 | **257.13** | 969.75 | **24.34** |
| ***CXCL2*** | <1.00 | 402.36 | **402.36** | 82.06 | **82.06** |
| ***TNFAIP3*** | <1.00 | 234.41 | **234.41** | 40.22 | **40.22** |
| ***CXCL10*** | 9.57 | 1342.15 | **140.28** | 156.56 | **16.36** |
| ***NFKBID*** | 1.00 | 100.43 | **100.43** | 92.33 | **92.33** |
| ***NFKBIZ*** | 33.33 | 2668.30 | **80.06** | 573.85 | **17.22** |
| ***TNF*** | <1 | 66.37 | **66.38** | 376.97 | **376.97** |
| ***ATF3*** | 4.51 | 283.06 | **62.82** | 53.25 | **11.82** |
| ***JUN*** | 20.55 | 1046.36 | **50.92** | 81.99 | **3.99** |
| ***GDF15*** | 37.73 | 1880.09 | **49.83** | 123.86 | **3.28** |
| ***RCAN1*** | 8.54 | 377.06 | **44.17** | 42.94 | **5.03** |
| ***IER3*** | 36.17 | 1593.77 | **44.07** | 325.58 | **9.00** |
| ***SAA3*** | 48.36 | 1105.92 | **22.87** | 65.94 | **1.36** |
| ***DSCR1*** | 91.48 | 1948.17 | **21.30** | 106.60 | **1.17** |
| ***CD83*** | 11.51 | 222.30 | **19.31** | 352.09 | **30.58** |
| ***GADD45B*** | 6.01 | 103.16 | **17.16** | 21.74 | **3.62** |
| ***ICAM1*** | 53.91 | 886.36 | **16.44** | 305.87 | **5.67** |
| ***IL1B*** | 62.18 | 974.33 | **15.67** | 1346.37 | **21.65** |
| ***NFKBIA*** | 90.39 | 1287.06 | **14.24** | 412.85 | **4.57** |
| ***JUNB*** | 68.11 | 921.17 | **13.52** | 497.52 | **7.30** |
| ***FOS*** | 7.53 | 87.83 | **11.66** | 322.09 | **42.77** |
| ***CCL4*** | 8.08 | 89.76 | **11.10** | 421.56 | **52.14** |
| ***TNFAIP2*** | 83.81 | 873.39 | **10.42** | 99.98 | **1.19** |
| ***AXUD1*** | 100.20 | 978.24 | **9.76** | 295.01 | **2.94** |
| ***MYD116*** | 49.88 | 482.49 | **9.67** | 379.69 | **7.61** |
| ***S100A9*** | 14.88 | 143.16 | **9.62** | 48.65 | **3.27** |
| ***PHLDA1*** | 306.86 | 2898.05 | **9.44** | 1469.56 | **4.79** |
| ***IRF1*** | 265.42 | 2419.74 | **9.12** | 401.73 | **1.51** |
| ***PLK3*** | 114.54 | 955.35 | **8.34** | 173.65 | **1.52** |
| ***BCL2A1B*** | 14.71 | 117.46 | **7.99** | 194.28 | **13.21** |
| ***TLR2*** | 33.02 | 261.29 | **7.91** | 74.97 | **2.27** |
| ***S100A8*** | 65.60 | 444.02 | **6.77** | 404.91 | **6.17** |
| ***ADRB2*** | 21.29 | 141.63 | **6.65** | 26.56 | **1.25** |
| ***ZFP36*** | 481.02 | 3146.39 | **6.54** | 1772.71 | **3.69** |
| ***IL1A*** | 18.27 | 115.64 | **6.33** | 247.43 | **13.54** |
| ***SERPINA7*** | 45.93 | 254.29 | **5.54** | 107.43 | **2.34** |
| ***MT-ND5*** | 61.85 | 318.63 | **5.15** | 6.48 | **0.10** |
| ***GADD45G*** | 82.61 | 383.62 | **4.64** | 90.08 | **1.09** |
| ***EGR1*** | 663.61 | 3001.22 | **4.52** | 1922.57 | **2.90** |
| ***CD14*** | 41.43 | 184.43 | **4.45** | 155.13 | **3.74** |
| ***CISH*** | 307.67 | 1365.51 | **4.44** | 242.40 | **0.79** |
| ***DUSP1*** | 249.58 | 960.73 | **3.85** | 1087.86 | **4.36** |
| ***SAA1*** | 365.95 | 1295.93 | **3.54** | 760.86 | **2.08** |
| ***PPP1R10*** | 108.04 | 378.08 | **3.50** | 212.79 | **1.97** |
| ***BCL3*** | 39.83 | 133.55 | **3.35** | 73.36 | **1.84** |
| ***SOCS3*** | 33.10 | 109.01 | **3.29** | 90.10 | **2.72** |
| ***BRD2*** | 218.63 | 711.63 | **3.25** | 350.69 | **1.60** |
| ***H2-EB1*** | 161.37 | 517.34 | **3.21** | 267.48 | **1.66** |
| ***COQ10B*** | 42.97 | 136.40 | **3.17** | 162.00 | **3.77** |
| ***CXCL9*** | 212.88 | 658.61 | **3.09** | 313.05 | **1.47** |
| ***H2-AB1*** | 319.92 | 963.66 | **3.01** | 547.18 | **1.71** |
| ***SLC25A25*** | 578.85 | 1695.90 | **2.93** | 1020.27 | **1.76** |
| ***DDIT4*** | 41.18 | 118.75 | **2.88** | 18.83 | **0.46** |
| ***HMOX1*** | 44.86 | 128.36 | **2.86** | 100.58 | **2.24** |
| ***CTGF*** | 53.83 | 146.43 | **2.72** | 155.49 | **2.89** |
| ***DNAJB1*** | 329.14 | 873.05 | **2.65** | 1229.72 | **3.74** |
| ***MYD88*** | 192.97 | 510.71 | **2.65** | 195.36 | **1.01** |
| ***VASN*** | 72.46 | 171.10 | **2.36** | 68.71 | **0.95** |
| ***FPR2*** | 62.12 | 141.30 | **2.27** | 69.36 | **1.12** |
| ***AKAP8L*** | 95.80 | 210.04 | **2.19** | 60.20 | **0.63** |
| ***IMPACT*** | 58.57 | 127.94 | **2.18** | 50.43 | **0.86** |
| ***BIK*** | 72.81 | 158.68 | **2.18** | 48.80 | **0.67** |
| ***KLF6*** | 55.75 | 120.43 | **2.16** | 80.15 | **1.44** |
| ***RAMP1*** | 52.49 | 113.28 | **2.16** | 46.11 | **0.88** |
| ***WSB1*** | 83.87 | 180.08 | **2.15** | 210.69 | **2.51** |
| ***RPL13A*** | 158.48 | 339.34 | **2.14** | 194.31 | **1.23** |
| ***SLC25A33*** | 199.50 | 423.00 | **2.12** | 545.02 | **2.73** |
| ***EFNA1*** | 103.79 | 217.33 | **2.09** | 65.28 | **0.63** |
| ***YRDC*** | 53.57 | 112.08 | **2.09** | 103.20 | **1.93** |
| ***GSTT2*** | 170.37 | 351.39 | **2.06** | 248.28 | **1.46** |
| ***PIM3*** | 417.14 | 857.63 | **2.06** | 399.07 | **0.96** |
| ***DLM1*** | 67.24 | 137.68 | **2.05** | 47.48 | **0.71** |
| ***MAT2A*** | 192.52 | 389.64 | **2.02** | 221.48 | **1.15** |
| ***GPIHBP1*** | 122.45 | 247.20 | **2.02** | 108.47 | **0.89** |
| ***TOB1*** | 317.93 | 639.01 | **2.01** | 601.05 | **1.89** |
| ***GIMAP6*** | 65.28 | 130.85 | **2.00** | 35.63 | **0.55** |
| ***LCN2*** | 444.66 | 890.22 | **2.00** | 611.72 | **1.38** |

Cutoff set to >100 signal and ≥2 fold increase in entolimod treated samples.
