# Supplementary material for: TLR5 agonist entolimod reduces the adverse toxicity of TNF while preserving its antitumor effects
Source: PLoS One. 2020 Feb 6;15(2):e0227940. doi: 10.1371/journal.pone.0227940 (PMC7004342; doi:10.1371/journal.pone.0227940)
Supplement: S2 Table — (DOCX) [file pone.0227940.s004.docx]

**S2 Table. Genes upregulated by entolimod in livers of NIH Swiss mice**

| **Gene** | **Untreated** | **Entolimod** | |
| --- | --- | --- | --- |
|  | **Mean** | **Mean** | **Fold change** |
| ***CXCL1*** | 9.12 | 124.05 | **13.60** |
| ***CXCL2*** | <1 | 150 | **150.00** |
| ***TNFAIP3*** | <1 | 207.83 | **207.83** |
| ***CXCL10*** | 1.4 | 573.42 | **409.59** |
| ***NFKBID*** | <1 | 43.14 | **43.14** |
| ***NFKBIZ*** | 22.76 | 1914.55 | **84.12** |
| ***TNF*** | <1 | 53.19 | **53.19** |
| ***ATF3*** | 3.73 | 144.57 | **38.76** |
| ***JUN*** | 32.97 | 756.734 | **22.95** |
| ***GDF15*** | 60.32 | 1558.36 | **25.83** |
| ***RCAN1*** | 50.84 | 456.37 | **8.98** |
| ***IER3*** | 17.15 | 739.62 | **43.13** |
| ***ICAM1*** | 55.64 | 272.11 | **4.89** |
| ***IL1B*** | 23.39 | 584.05 | **24.97** |
| ***NFKBIA*** | 236.78 | 1198.51 | **5.06** |
| ***JUNB*** | 70.25 | 531.47 | **7.57** |
| ***FOS*** | <1 | 142.16 | **142.16** |
| ***CCL4*** | <1 | 59.28 | **59.28** |
| ***TNFAIP2*** | 49.47 | 394.93 | **7.98** |
| ***MYD116*** | 67.4 | 291.62 | **4.33** |
| ***IRF1*** | 109.08 | 1347.48 | **12.35** |
| ***BCL2A1B*** | 18.47 | 51.44 | **2.79** |
| ***ADRB2*** | 64.35 | 137.97 | **2.14** |
| ***IL1A*** | 6.85 | 86.58 | **12.64** |
| ***MT-ND5*** | 80.58 | 275.95 | **3.42** |
| ***SOCS3*** | 16.48 | 48.59 | **2.95** |
| ***CXCL9*** | 73.67 | 461.47 | **6.26** |
| ***DLM1*** | 7.68 | 27.53 | **3.58** |

Cutoff set to >100 signal and ≥2 fold increase in the treated samples.
