# Supplementary material for: TLR5 agonist entolimod reduces the adverse toxicity of TNF while preserving its antitumor effects
Source: PLoS One. 2020 Feb 6;15(2):e0227940. doi: 10.1371/journal.pone.0227940 (PMC7004342; doi:10.1371/journal.pone.0227940)
Supplement: S3 Table — (DOCX) [file pone.0227940.s005.docx]

**S3 Table. Genes upregulated in entolimod-treated cultured hepatocytes**

| **Gene** | **Untreated** | **Entolimod** | |
| --- | --- | --- | --- |
|  | **Mean** | **Mean** | **Fold induction** |
| ***CCL20*** | <1.00 | 353.10 | **353.10** |
| ***CXCL2*** | 4.90 | 1503.60 | **306.86** |
| ***CSF2*** | 47.30 | 1563.40 | **33.05** |
| ***VCAM1*** | 18.50 | 342.70 | **18.52** |
| ***NFKBIZ*** | 367.20 | 5354.90 | **14.58** |
| ***TNFAIP3*** | 115.40 | 1604.10 | **13.90** |
| ***NFKBID*** | 61.80 | 698.60 | **11.30** |
| ***IER3*** | 1795.60 | 17678.40 | **9.85** |
| ***LIF*** | 63.20 | 511.50 | **8.09** |
| ***PDE4B*** | 15.80 | 113.30 | **7.17** |
| ***TNFAIP2*** | 42.50 | 303.70 | **7.15** |
| ***IRF1*** | 566.90 | 4046.10 | **7.14** |
| ***EDN1*** | 122.20 | 793.40 | **6.49** |
| ***NFKBIA*** | 720.60 | 4531.00 | **6.29** |
| ***ICAM1*** | 460.80 | 2456.80 | **5.33** |
| ***CXCL1*** | 3909.90 | 19604.70 | **5.01** |
| ***CCL2*** | 598.20 | 2981.90 | **4.98** |
| ***CISH*** | 125.20 | 616.10 | **4.92** |
| ***DUSP8*** | 86.80 | 406.10 | **4.68** |
| ***PLSCR5*** | 29.70 | 132.90 | **4.47** |
| ***CCL7*** | 42.70 | 186.40 | **4.37** |
| ***CSRNP1*** | 56.90 | 222.50 | **3.91** |
| ***SLC25A25*** | 263.40 | 1003.20 | **3.81** |
| ***DUSP8*** | 118.80 | 444.40 | **3.74** |
| ***ZFP36*** | 368.30 | 1365.00 | **3.71** |
| ***SAA3*** | 132.30 | 485.10 | **3.67** |
| ***PHLDA1*** | 1526.60 | 5564.30 | **3.64** |
| ***JUN*** | 72.10 | 254.00 | **3.52** |
| ***TLR2*** | 127.20 | 425.30 | **3.34** |
| ***ADRB2*** | 195.70 | 634.80 | **3.24** |
| ***GADD45B*** | 195.60 | 633.90 | **3.24** |
| ***RCAN1*** | 163.30 | 525.60 | **3.22** |
| ***ATF3*** | 167.60 | 533.30 | **3.18** |
| ***CSF1*** | 192.40 | 583.40 | **3.03** |
| ***JUNB*** | 950.60 | 2681.90 | **2.82** |
| ***RHOB*** | 162.80 | 454.10 | **2.79** |
| ***ZFP36L1*** | 952.50 | 2539.10 | **2.67** |
| ***MYD116*** | 201.20 | 533.60 | **2.65** |
| ***TNRC6C*** | 45.00 | 115.10 | **2.56** |
| ***RCAN1*** | 383.70 | 965.50 | **2.52** |
| ***GDF15*** | 343.10 | 848.30 | **2.47** |
| ***CYR61*** | 229.30 | 566.50 | **2.47** |
| ***BCL2L11*** | 129.00 | 299.20 | **2.32** |
| ***AREG*** | 687.30 | 1575.80 | **2.29** |
| ***PLK3*** | 398.00 | 892.90 | **2.24** |
| ***PVR*** | 138.20 | 304.10 | **2.20** |
| ***ANKRD1*** | 4108.40 | 8713.70 | **2.12** |
| ***CEP120*** | 61.50 | 124.70 | **2.03** |
| ***TBX1*** | 54.80 | 110.00 | **2.01** |

Cutoff set to >100 signal and ≥2 fold increase in the treated samples.
